# Factors influencing quality of life among married nurses with children in Korea: a cross-sectional survey

**DOI:** 10.4069/whn.2025.05.20

**Published:** 2025-09-30

**Authors:** Yunmi Kim

**Affiliations:** College of Nursing, Jesus University, Jeonju, Korea

**Keywords:** Health behavior, Lifestyle, Personal autonomy, Psychological stress, Quality of life

## Abstract

**Purpose:**

This study aimed to identify factors influencing quality of life (QoL) among married nurses with children working in Korea, with a focus on self-determination (SDT), health-promoting lifestyle (HPL), job satisfaction, and parenting stress.

**Methods:**

A cross-sectional design was utilized, involving 150 married nurses with children recruited from three general hospitals in Jeonju, Korea. Data were collected between July 30 and November 31, 2023. Variables measured included HPL, SDT, job satisfaction, parenting stress, and QoL. Data analysis comprised descriptive statistics, the t-test, Pearson correlation coefficients, and multiple regression analysis.

**Results:**

The mean QoL score was 77.67±12.75 on a scale of 26–130, indicating a moderate level. HPL was identified as the strongest positive predictor (β=.49, *p*<.001), followed by SDT (β=.21, *p*=.003). Parenting stress showed a significant negative association with QoL (β=–.15, *p*=.049). Together, these variables explained 52.6% of the variance in QoL (F=16.18, *p*<.001). Other variables, including job satisfaction, primary caregiver support, spousal support, turnover intention, and having a child with a chronic illness, were not statistically significant in the final model.

**Conclusion:**

HPL, SDT, and parenting stress significantly influenced QoL in this population, with HPL being the most influential factor. These findings suggest that interventions targeting health-promoting behaviors and SDT, while reducing parenting stress, may be critical for improving the QoL of married nurses with children working in general hospitals.

## Introduction

The average age of nurses working in Korean general and tertiary hospitals is approximately 33.4 years, suggesting a potential future decline in the active nursing workforce due to retirement and aging [1]. This concern has prompted numerous studies on the retention of experienced married nurses, underscoring the importance of understanding how these nurses balance professional obligations with family responsibilities [2,3]. Furthermore, the coronavirus disease 2019 (COVID-19) pandemic has heightened interest in the quality of life (QoL) of married nurses [4]. Despite growing attention to nurse well-being, particularly since the pandemic, there remains a scarcity of research investigating the complex factors influencing the QoL of married nurses [4,5].

Married nurses often encounter greater challenges managing dual roles at work and at home [5,6]. Although several studies have examined the QoL of nurses in general, there is limited understanding of the specific factors affecting QoL among married nurses in Korea. While prior research has addressed various perspectives on nurses’ QoL [7,8], studies focused on married nurses have primarily explored aspects such as lifestyle [9], physical health [10], job satisfaction [11], parenting stress [12,13], and family–work conflict [3,13].

Recent research consistently demonstrates that self-determination is positively associated with psychological stability and life satisfaction [4,14]. For instance, higher self-determination has been shown to improve life satisfaction among college students facing adjustment difficulties [4]. Moreover, self-determination is recognized as a predictor of psychological well-being, which is an essential component of QoL [8,15].

Health-promoting behaviors (HPBs) are vital for sustaining well-being, managing stress, and enhancing the QoL of married nurses, who frequently encounter unique challenges in balancing work and family life. According to Pender’s Health Promotion Model, individuals actively engage in HPBs shaped by personal, social, and environmental factors [16]. These behaviors not only promote physical health but also contribute to emotional resilience and increased job satisfaction—both of which are especially important in the nursing profession.

Married nurses with children commonly experience role conflict and parenting stress, which can significantly impact their job satisfaction and overall QoL [3,17]. As married nurses manage dual responsibilities at home and work, increased role conflict is associated with decreased job satisfaction and QoL [3]. Notably, conflict between professional and domestic spheres remains a major stressor closely linked to reduced QoL [3,12]. From the perspective of self-determination theory (SDT), parenting stress should thus be regarded as a key variable substantially influencing QoL [8,15]. Addressing parenting stress and role conflict is especially important for married nurses in their 30s and 40s, as these factors affect both their personal well-being [3,18] and job satisfaction [17].

Job satisfaction is crucial for enabling nurses to effectively apply their skills to achieve professional objectives and improve their QoL; it also plays a key role in increasing retention, thereby preserving the expertise of seasoned nurses within healthcare organizations [11]. Given the aging nursing workforce, urgent strategies are needed to strengthen retention and safeguard the accumulated experience of senior nurses [2,3].

Identifying core factors such as self-determination, health-promoting lifestyle (HPL), job satisfaction, and parenting stress is essential for designing institution-wide interventions and supportive policies to enhance the QoL of married nurses with children. By examining the distinct challenges these nurses face in reconciling professional and personal responsibilities, this study aims to provide foundational insights for more effective support. Addressing these issues will empower nurses to achieve a healthier work–life balance and offer actionable data to promote their well-being.

### Research objectives

This study aimed to examine the effects of self-determination, HPL, job satisfaction, and parenting stress on the QoL of married nurses with children.

## Methods

**Ethics statement**

This study was conducted following approval from the Institutional Review Board of Jesus Hospital (2023-06-018). All participants voluntarily provided informed consent after receiving a full explanation of the study’s purpose.

### Study design and participants

A descriptive correlational design with a cross-sectional survey was used to investigate factors associated with married female nurses with children. The study was reported in accordance with the STROBE guidelines (https://www.strobe-statement.org/).

Participants were recruited based on the following selection criteria: (1) being a married female nurse with children, (2) employment at a general hospital with more than 500 beds, and (3) a full understanding of the study’s purpose and voluntary written consent to participate. Male nurses were excluded.

The target sample size was calculated using G*Power 3.1.9.7 [19]. With a significance level of .05, an effect size of .15, and a power of .80—parameters based on previous research [17,20]—multiple regression analysis with 19 predictor variables indicated a minimum sample size of 153. Allowing for a potential dropout rate of approximately 20% [3,20], a total of 190 questionnaires were distributed. Of these, 160 were returned (response rate, 84.2%), and after excluding 10 incomplete responses, 150 questionnaires were included in the final analysis. The actual dropout rate was 21.1%, which was consistent with expectations and within acceptable limits.

### Measurements

The use of all measurement instruments in this study was approved by their respective developers and/or translators.

#### Quality of life

QoL was assessed using the World Health Organization Quality of Life assessment instrument (WHOQOL-BREF), derived from the WHOQOL-100 [21] and standardized in Korean by Min et al. [22]. This instrument consists of 26 items, including two general items measuring overall QoL and health status, and four domain-specific subscales: physical health (possible range, 7–35), psychological health (possible range, 6–30), social relationships (possible range, 3–15), and environment (possible range, 8–40). Each item is rated on a 5-point Likert scale (1, not at all to 5, very much). Some items are reverse-scored, and the total score ranges from 26 to 130, with higher scores indicating better QoL. Cronbach’s α was .96 at the time of original development [21], .89 for the Korean version [22], and .91 in this study.

#### Self-determination

For self-determination, the Basic Psychological Needs Scale (BPNS) developed by Deci and Ryan [15] and translated and validated in Korean by Lee and Kim [23] was used. This scale assesses three core psychological needs as proposed in SDT: autonomy, competence, and relatedness. It consists of 18 items, with six items for each subdomain, rated on a 5-point Likert scale (1, not at all to 5, very much). Higher total scores (possible range, 18–90) indicate greater self-determination, with subdomain score ranges as follows: autonomy (possible range, 6–30), competence (possible range, 6–30), and relatedness (possible range, 6–30). The BPNS has been widely applied to measure self-determination and QoL across various populations and cultural settings [5,14]. The Cronbach’s α values at the time of tool development [23] were .84 for autonomy, .88 for competence, and .86 for relatedness. In this study, overall reliability was .85, with subdomain reliabilities of .77 (autonomy), .83 (competence), and .75 (relatedness).

#### Health-promoting lifestyle

HPBs were assessed using the Korean version [24] of the Health Promoting Lifestyle Profile II, originally developed by Walker et al. [16]. The instrument consists of 52 items, each rated on a 4-point Likert scale (1, not at all to 4, regularly), with higher scores (range, 52–208) indicating a greater degree of HPB. It comprises six subdomains with the following score ranges: spiritual growth (9–36), physical activity (8–32), health responsibility (9–36), interpersonal relations (9–36), nutrition (9–36), and stress management (8–32). Cronbach’s was .94 for the original scale [24] and .95 in this study (with subdomain reliabilities ranging from .70 to .86).

#### Job satisfaction

Job satisfaction was measured using the Korean version [25] of the instrument developed by Slavitt et al. [26]. The tool comprises 29 items, each rated on a 5-point Likert scale (range, 1–5), with higher scores (range, 29–145) representing greater job satisfaction. The scale includes five subdomains: pay, administrative requirements, professionalism, autonomy, and job demands. Nine negatively worded items were reverse-scored. Cronbach’s α was .72 at original development [26], .83 among Korean nurses [25], and .73 in this study.

#### Parenting stress

Parenting stress was measured using the Korean Parenting Stress Index-Short Form, 4th edition (K-PSI-4-SF), which is based on the original Parenting Stress Index Short Form, 4th edition (PSI-SF-4) [27] and was translated, validated, and purchased for this study (https://inpsyt.co.kr/psy/item/view/KPSI4_CO_PG) [28]. The PSI-SF-4 contains 36 items in three subdomains: parental distress, parent-child dysfunctional interaction, and difficult child. Each item is rated on a 5-point Likert scale (1, strongly disagree, to 5, strongly agree), with higher summed scores (range, 36–180) reflecting greater parenting stress. The Cronbach’s α for the K-PSI-4-SF was .96 [28] and .92 in this study.

### Study procedures

This study was conducted between July 30 and November 31, 2023, and targeted married nurses with children employed at three general hospitals in Jeonju, Korea.

Data collection began after receiving permission from the head of the nursing department. The researcher explained the purpose and procedures of the study and obtained informed consent from all participants. Questionnaires were distributed in individual envelopes to eligible nurses using the snowball sampling method and were collected via the nursing department. Upon completion, participants received a small gift (a mobile gift certificate worth approximately 4 US dollars [USD]).

### Statistical analysis

Data were analyzed using IBM SPSS Statistics for Windows, ver. 26.0 (IBM Corp., Armonk, NY, USA). Descriptive statistics (frequencies, percentages, means, and standard deviations) were used to summarize demographic characteristics and main variables. To examine differences in QoL according to demographic characteristics, the independent-samples t-test and analysis of variance were conducted, with Scheffé post hoc tests applied as appropriate. Pearson correlation coefficients were calculated to assess relationships among study variables, and normality was evaluated using skewness and kurtosis. Instrument reliability was determined with Cronbach’s alpha. Statistical significance was set at *p*=.05.

Multiple regression analysis was performed to identify factors influencing QoL, using a forced-entry method in which all independent variables were entered simultaneously [29]. This approach enabled a comprehensive assessment of how demographic and previously identified factors—such as primary support, spouse support, turnover intention, children’s chronic illness [9], HPL [4,22], job satisfaction [2], parenting stress [13], and core SDT variables—explained QoL. By including all predictors at once, we could evaluate the combined explanatory power of these variables and control for their interrelationships [30].

## Results

### General characteristics of participants

The average age of participants was 41.1 years, and 50.0% were between 31 and 49 years old. The majority of spouses were also between 31 and 49 years old (57.3%). In terms of education, 56.7% of participants held a university degree, while among spouses, 64.7% had completed university. Regarding support satisfaction, 54.0% of participants reported satisfaction with primary caregiver support, 40.0% with workplace peer support, and 56.7% with spouse support. Additionally, 65.3% of participants expressed turnover intention, whereas 34.7% did not intend to leave their positions. With respect to night shifts, 66.7% did not work night shifts, 8.7% worked fewer than three times per month, and 24.7% worked more than four times per month. Clinical experience varied, with 28.7% having 10 to 14 years of experience and 58.6% having 15 or more years. Most participants were staff nurses (76.7%), followed by charge nurses (14.7%), and head nurses or those in other managerial positions (8.6%). During the data collection period in 2023, a majority of participants (87.3%) reported a monthly personal income of 3 million Korean won (KRW; 2,308 USD) or higher. While this exceeds South Korea’s 2023 median individual monthly wage of 3.5 million KRW (2,692 USD), it remains below the national average household income of 5.03 million KRW (3,869 USD) in Q3 2023—a statistic that aggregates all earners within a family. As the survey captured individual wages, the 3 million KRW threshold was retained, representing the upper-middle band of personal earnings for Korean nurses. Additionally, 43.6% of participants reported alcohol consumption, and 11.3% had children with chronic illnesses (e.g., those requiring long-term treatment for over 6 months). Participants were raising an average of 1.88 children, with 27.3% having one child, 59.3% having two children, and 13.4% having three or more children (Table 1).

### Participants’ levels of quality of life, self-determination, health-promoting lifestyle, job satisfaction, and parenting stress

Participants reported generally favorable scores across all measured domains (Table 2). Overall QoL was 77.67±12.75, a mid-to-upper-range value, with subdomain scores indicating moderate well-being in physical (19.21±3.31), psychological (17.73±3.20), social (9.22±2.17), and environmental (24.97±4.80) dimensions. Basic psychological needs were satisfied at a moderately high level (79.27±8.14), supported by relatively strong autonomy (25.03±4.53), competence (26.45±3.44), and relatedness (27.81±2.63). Engagement in a HPL was moderate overall (122.27±21.60). Scores were highest for interpersonal relations (24.86±3.98) and spiritual growth (23.06±4.89), but lowest for physical activity (15.61±5.36), indicating room for improvement in exercise habits. Job satisfaction was in the upper-mid range (85.91±10.95), while parenting stress averaged 74.68±16.86—moderate on average, although some nurses experienced elevated stress. All distributions exhibited acceptable skewness and kurtosis, with values within 2, indicating approximate normality for all variables.

### Differences in quality of life according to general characteristics

Analysis of QoL differences based on general characteristics revealed that participants satisfied with primary caregiver support (F=8.56, *p*=.001), workplace peer support (F=9.09, *p*=.001), and spouse support (F=8.79, *p*=.001) had significantly higher QoL than those who were dissatisfied. Furthermore, participants without turnover intention (t=–2.02, *p*=.045) and those whose children did not have chronic illnesses (t=–2.52, *p*=.021) reported significantly higher QoL (Table 1).

### Relationships among quality of life, self-determination, health-promoting lifestyle, job satisfaction, and parenting stress factors

As shown in Table 3, QoL was significantly positively correlated with self-determination (r=.50, *p*=.001), HPL (r=.61, *p*=.001), and job satisfaction (r=.23, *p*=.004), and significantly negatively correlated with parenting stress (r=–.44, *p*=.001). Among subdomains, stress management, interpersonal relations, and spiritual growth were most strongly related to QoL.

Self-determination was positively correlated with both HPL and job satisfaction, and negatively correlated with parenting stress. These findings indicate that greater self-determination, engagement in HPBs, and higher job satisfaction are associated with better QoL, whereas higher parenting stress is linked to lower QoL (Table 3).

### Factors influencing the quality of life of married nurses with children working in general hospitals

As indicated in Table 4, the final regression model significantly predicted QoL (F=16.18, *p*=.001), explaining 52.6% of the variance. In this model, HPL emerged as the strongest positive predictor (β=.49, *p*=.001), followed by self-determination (β=.21, *p*=.003). Parenting stress had a significant negative effect on QoL (β=–.15, *p*=.049), suggesting that lower stress levels are associated with higher QoL.

Other variables—including primary caregiver support, spouse support, turnover intention, and having a child with a chronic illness—were not statistically significant predictors in the model. The Durbin-Watson statistic was 1.908, and variance inflation factor values ranged from 1.132 to 10.692, indicating no serious issues with autocorrelation or multicollinearity.

## Discussion

This study found a moderate overall level of QoL among married nurses with children (77.67±12.75). Compared to a previous study on nurses in tertiary general hospitals (3.19–3.41 on a 1–5 scale) [6], this level is relatively low (point average of 2.98 on a 1–5 scale), suggesting that balancing professional and parental roles may present additional challenges in general hospital settings.

Among the factors examined, HPL emerged as the most significant predictor of QoL, with participants reporting moderate engagement in HPBs. Subdomain analysis indicated that interpersonal relations had the highest average (24.9), reflecting the importance of strong social networks both at work and at home. In contrast, Physical Activity was notably low (15.6), likely due to shift work, heavy workloads, and childcare responsibilities [9,18]. Nurses aged 40 years or older tended to maintain healthier lifestyles, possibly due to greater health awareness and accumulated life experience [9]. Nonetheless, consistent physical activity remained a challenge for those balancing shift work and caregiving responsibilities [17]. Previous studies suggest that capacity-building efforts, such as continuing education, mentoring, and family-support programs, can enhance HPBs and reduce burnout among married nurses [9,15]. Therefore, structured physical activity initiatives, flexible scheduling, and institutional family support are recommended to address these barriers and promote sustained health behaviors.

Self-determination was also a significant positive predictor of QoL in our regression analysis. The high average score of 81.44 on the BPNS (range, 18–90) indicates that autonomy, competence, and relatedness needs were generally well met. Importantly, after controlling for other factors, higher need satisfaction remained independently associated with better QoL, supporting SDT’s central assertion that fulfilling these psychological needs enhances well-being [15]. Nurses who experience decision-making freedom, such as participation in work-related decisions or negotiating shift patterns, report lower stress and higher professional QoL. In studies of nurses in general hospitals in Korea [8] and among shift-working nurses [20], autonomy was identified as a major predictor of work-related QoL. Nevertheless, unpredictable shift work continues to exacerbate work–family imbalance and childcare stress among married nurses, thereby diminishing overall QoL [17].

Parental stress is a key factor in reducing the QoL of married nurses, and its influence has been consistently demonstrated in previous studies as well as in the present findings. In particular, a study of married nurses working shifts showed a strong negative correlation between parenting stress and QoL, and when combined with role conflict, the explanatory power increased to 53% [17]. These findings indicate that the burden of childcare affects both work performance and emotional stability. During the COVID-19 pandemic, parenting stress increased by 47% [3], and nurses with children needing care reported the lowest QoL [17]. This is attributed to a lack of time for childcare caused by unpredictable shift patterns, which heighten stress. Notably, 65.3% of nurses in general hospitals in Korea cited “inability to balance work and family life” as a reason for considering leaving their jobs [12], demonstrating that parental stress can lead to job turnover. Organizational strategies are essential to mitigate parental stress. Actual spousal involvement in childcare reduced stress by 34% [13], while employee support systems improved QoL by reducing work–family conflict by 20% [31]. Additionally, the introduction of flexible working hours was found to improve job satisfaction by reducing role conflict by 0.26 standard deviations [11]. These results highlight the importance of family-friendly policies and the development of social support networks in managing parental stress.

Furthermore, strong relatedness, manifested through supportive colleagues at work and spousal assistance at home, has been shown to enhance nurses’ QoL; when these sources of support operate together, they buffer stress and improve overall well-being [8,13]. In contrast, increased parenting stress was associated with significantly lower QoL, a finding consistent with studies of married nurses working shifts in general hospitals in Korea, which found that nurses caring for children requiring long-term care had the lowest QoL [17]. This added care burden can result in emotional and financial strain. For nurses with children, the conflict between work and family life—particularly the lack of time for childcare due to unpredictable shifts—further exacerbates this burden. In a survey of nurses in general hospitals in Korea, 65.3% reported that they were considering job changes for this very reason [11,12]. These results suggest that organizational measures such as in-house childcare, childcare subsidies, and flexible working hours are needed to help nurses balance their professional and domestic roles and reduce parenting stress.

Although job satisfaction was not a significant predictor of QoL in this study, this does not diminish its potential influence on nurses’ well-being. Previous research indicates that low job satisfaction can adversely affect both physical and mental health [6,11]. Meanwhile, practical support from primary caregivers or spouses proved beneficial: nurses who received consistent, hands-on assistance at home reported reduced parenting stress and improved QoL [7,13]. Positive social relationships and cooperative work environments also contributed to better well-being; however, irregular shift patterns frequently hinder the formation or maintenance of these supportive connections [17,20].

Therefore, this study has several limitations. First, the key predictors—HPL, self-determination (autonomy, competence, relatedness), and parenting stress—were all assessed via self-report measures, which may introduce social desirability and recall biases. Future research should incorporate objective assessments (e.g., wearable health monitors, ecological momentary assessment via digital diaries) to capture real-time behaviors and stress fluctuations. Second, this study did not fully examine several factors known to influence nurses’ psychological well-being and QoL, including organizational culture [8], sleep quality [7,20], workplace stress [5], leadership support, and shift work [17,20]. Incorporating these variables in subsequent investigations could yield a more comprehensive understanding of QoL determinants among married nurses with children. Third, data were collected using convenience sampling, and a substantial proportion of participants held a university degree or higher (83.4%), with 26.7% having attained a master’s degree or above. This suggests that the sample may be skewed toward highly educated nurses, which may not accurately represent the broader population of married nurses with children in Korea. Similarly, the majority of participants reported relatively high monthly incomes (87.3% earning 3 million KRW or more), reflecting a socioeconomic status higher than the national average for nurses. This overrepresentation of highly educated and high-income individuals may have influenced the self-reported HPL, stress, and QoL outcomes, and may not accurately reflect the broader population of married nurses with children in Korea. Although higher income has been consistently associated with better QoL among nurses, the impact of education level on QoL is less clear and may not be significant in all contexts. Fourth, the sample was predominantly composed of staff nurses (76.7%), with relatively few participants in managerial or advanced roles, which may limit the generalizability of these findings to nursing leaders who face distinct work–life balance challenges and stressors [32].

In conclusion, this study demonstrated that, among married nurses with children, QoL is most strongly and positively associated with HPL, followed by self-determination (autonomy, competence, and relatedness), and is inversely associated with parenting stress. Targeted interventions, such as programs that encourage regular engagement in HPBs and strengthen psychological need satisfaction, are likely to yield the greatest improvements in QoL. Furthermore, implementing flexible work policies, peer support initiatives, and family-friendly measures to reduce parenting stress and childcare burdens may further empower this group, ultimately enhancing their QoL and promoting a more sustainable nursing workforce.

## Figures and Tables

**Table 1.** General characteristics of participants (N=150)

Variable	Categories	Mean±SD or n (%)	Quality of life	
			Mean±SD	t/F (*p*) Scheffé
Age (year)		41.09±5.91		
	≤30	60 (40.0)	77.15±12.76	0.17 (.847)
	31–49	75 (50.0)	77.76±12.94	
	≥50	15 (10.0)	79.27±12.46	
Spouse age (year)		43.54±5.90		
	≤30	38 (25.3)	78.86±12.76	0.57 (.567)
	31–49	86 (57.3)	76.71±13.10	
	≥50	26 (17.4)	79.12±11.72	
Education level	College	25 (16.6)	74.27±10.41	1.51 (.224)
	University	85 (56.7)	77.21±13.17	
	≥Master	40 (26.7)	80.03±12.96	
Spouse education level	≤College	29 (19.3)	72.75±12.44	2.81 (.064)
	University	97 (64.7)	78.48±13.35	
	≥Master	24 (16.0)	80.17±9.67	
Primary caregiver support	Satisfied^a^	81 (54.0)	80.85±11.86	8.56 (<.001) a,b>c
	Neutral^b^	62 (41.3)	74.93±12.50	
	Dissatisfied^c^	7 (4.7)	64.29±12.35	
Workplace peer support	Satisfied^a^	60 (40.0)	82.64±12.59	9.09 (<.001) a>c
	Neutral^b^	87 (58.0)	74.67±11.68	
	Dissatisfied^c^	3 (2.0)	65.67±14.84	
Spouse support	Satisfied^a^	85 (56.7)	81.19±11.61	8.79 (<.001) a>c
	Neutral^b^	51 (34.0)	73.92±13.06	
	Dissatisfied^c^	14 (9.3)	69.93±11.72	
Turnover intention	Yes	98 (65.3)	76.34±12.91	–2.02 (.045)
	No	52 (34.7)	80.74±11.66	
Night shift	None	100 (66.7)	78.68±11.93	1.07 (.347)
	1–3	13 (8.7)	74.08±14.24	
	≥4	37 (24.7)	78.68±11.93	
Total clinical experience (year)	<10	19 (12.7)	80.84±14.96	0.70 (.497)
	10–14	43 (28.7)	76.81±12.78	
	≥15	88 (58.6)	77.39±12.27	
Position	Staff nurse	115 (76.7)	77.40±13.01	0.93 (.428)
	Charge nurse	22 (14.7)	76.32±13.26	
	Head nurse, etc.	13 (8.6)	80.60±8.97	
Monthly income (KRW)	<3 million	19 (12.7)	80.95±8.95	1.47 (.151)
	≥3 million	131 (87.3)	77.15±13.17	
Alcohol consumption	Yes	65 (43.6)	76.88±12.00	–0.68 (.495)
	No	85 (56.4)	78.31±13.41	
Child(ren) with chronic illness	Yes	17 (11.3)	70.12±13.25	–2.52 (.021)
	No	133 (88.7)	78.65±12.40	
Number of children		1.88±0.68		
	1	41 (27.3)	76.63±12.73	0.21 (.808)
	2	89 (59.3)	78.21±13.22	
	≥3	20 (13.4)	77.45±11.06	

KRW, Korean won (1 million KRW is approximately 770 US dollars).

**Table 2.** QoL, SDT, HPL, job satisfaction, and parenting stress levels (N=150)

Variable	Mean±SD	Min	Max	Possible range	Skewness		Kurtosis	
					Statistic	SE	Statistic	SE
QoL	77.67±12.75	45.00	115.00	26–130	–0.05	0.20	–0.16	0.40
Overall QoL	6.55±1.49	3.00	10.00	2–10	–0.20	0.20	–0.22	0.39
Physical health	19.21±3.31	12.00	28.00	7–35	–0.15	0.20	–0.30	0.40
Psychological health	17.73±3.20	8.00	28.00	6–30	0.10	0.20	0.18	0.39
Social relationships	9.22±2.17	4.00	15.00	3–15	–0.06	0.20	–0.05	0.39
Environment	24.97±4.80	13.00	36.00	8–40	–0.17	0.20	–0.41	0.40
SDT	79.27±8.14	42.00	90.00	3–90	–1.06	0.20	2.03	0.40
Autonomy	25.03±4.53	13.00	30.00	1–30	–0.64	0.20	–0.51	0.40
Competence	26.45±3.44	6.00	30.00	1–30	–2.14	0.20	0.33	0.39
Relatedness	27.81±2.63	20.00	30.00	1–30	–1.06	0.20	–0.08	0.39
HPL	122.27±21.60	75.00	191.00	52–208	0.46	0.20	0.27	0.40
Health responsibility	19.79±4.36	11.00	32.00	9–36	0.32	0.20	–0.25	0.40
Physical activity	15.61±5.36	8.00	32.00	8–32	0.63	0.20	0.20	0.39
Nutrition	20.68±3.69	12.00	33.00	9–36	0.50	0.20	0.49	0.40
Stress management	18.41±4.12	10.00	31.00	8–36	0.41	0.20	–0.14	0.40
Interpersonal relations	24.86±3.98	15.00	36.00	9–36	0.07	0.20	–0.01	0.40
Spiritual growth	23.06±4.89	10.00	36.00	9–36	0.13	0.20	–0.11	0.40
Job satisfaction	85.91±10.95	43.00	116.00	29–145	–0.18	0.20	1.53	0.39
Parenting stress	74.68±16.86	40.00	146.00	36–180	0.53	0.20	1.16	0.40

HPL, Health-promoting lifestyle; QoL, quality of life; SDT, self-determination theory.

**Table 3.** Relationships among QoL, SDT, HPL, job satisfaction, and parenting stress (N=150)

Variable	r (*p*)												
	QoL	SDT				HPL							Job satisfaction
		Total	1	2	3	Total	4	5	6	7	8	9	
QoL	1												
SDT (total)	.50 (<.001)	1											
1. Autonomy	.38E (<.001)	.86 (<.001)	1										
2. Competence	.40 (<.001)	.73 (<.001)	.39 (<.001)	1									
3. Relatedness	.38 (<.001)	.67 (<.001)	.42 (<.001)	.26 (.001)	1								
HPL (total)	.61 (<.001)	.38 (<.001)	.24 (.005)	.39 (<.001)	.26 (.002)	1							
4. Health responsibility	.37 (<.001)	.16 (.058)	.03 (.700)	.22 (<.001)	.15 (.076)	.81 (<.001)	1						
5. Physical activity	.41 (<.001)	.21 (.009)	.10 (.211)	.31 (<.001)	.09 (.285)	.83 (<.001)	.64 (<.001)	1					
6. Nutrition	.38 (<.001)	.27 (.001)	.13 (.105)	.35 (<.001)	.17 (.040)	.76 (<.001)	.63 (<.001)	.57 (<.001)	1				
7. Stress management	.52 (<.001)	.34 (<.001)	.28 (.001)	.32 (<.001)	.18 (.033)	.86 (<.001)	.58 (<.001)	.71 (<.001)	.59 (<.001)	1			
8. Interpersonal relations	.58 (<.001)	.44 (<.001)	.30 (<.001)	.27 (.001)	.51 (<.001)	.78 (<.001)	.57 (<.001)	.48 (<.001)	.47 (<.001)	.60 (<.001)	1		
9. Spiritual growth	.61 (<.001)	.45 (<.001)	.31 (<.001)	.43 (<.001)	.30 (<.001)	.84 (<.001)	.55 (<.001)	.54 (<.001)	.53 (<.001)	.73 (<.001)	.73 (<.001)	1	
Job satisfaction	.23 (.004)	.36 (<.001)	.36 (<.001)	.17 (.034)	.27 (.001)	.26 (.002)	.08 (.358)	.16 (.056)	.19 (.021)	.19 (.019)	.34 (<.001)	.24 (.003)	1
Parenting stress	–.44 (<.001)	–.39 (<.001)	–.45 (<.001)	–.13 (.117)	–.24 (.003)	–.23 (.007)	–.00 (.961)	–.11 (.182)	–.12 (.164)	–.25 (.003)	–.33 (<.001)	–.29 (<.001)	–.34 (<.001)

HPL, Health-promoting lifestyle; QoL, quality of life; SDT, self-determination theory.

**Table 4.** Factors influencing quality of life (N=150)

Factor	B	SE	β	t	*p*	95%CI		VIF
						Lower	Upper	
(Constant)	23.960	1.97		12.17	<.001	20.065	27.855	
Primary Support	4.05	4.85	0.16	0.84	.405	–5.539	13.639	10.692
Satisfied^†^								
Neutral^†^	3.80	4.57	0.15	0.83	.407	–5.236	12.836	9.256
Spouse support								
Satisfied^†^	2.28	3.40	0.09	0.67	.505	–4.442	9.002	5.239
Neutral^†^	–0.64	3.23	–0.02	–0.20	.843	–7.026	5.746	4.303
Turnover intention (Yes)^†^	–0.57	1.67	–0.02	–0.34	.736	–3.872	2.732	1.145
Chronic illness children (Yes)^†^	–4.07	2.45	–0.10	–1.66	.099	–8.914	0.774	1.132
HPL	0.29	0.04	0.49	7.53	<.001	0.211	0.369	1.227
Job satisfaction	–0.03	0.08	–0.03	–0.40	.693	–0.188	0.128	1.290
Parenting stress	–0.11	0.06	–0.15	–1.98	.049	–0.229	0.009	1.594
SDT	0.33	0.11	0.21	3.03	.003	0.113	0.547	1.421
Adjusted R^2^ (p)	.526 (<.001)							
F(p)	16.18(<.001)							

B, Unstandardized coefficient; β, standardized coefficient; CI, confidence interval; VIF, variance inflation factor.

† The reference variables were primary support (dissatisfied), spouse support (dissatisfied), turnover intention (no), and chronic illness (no).
